# Understanding and Managing Zoonotic Risk in the New Livestock Industries

**DOI:** 10.1289/ehp.1206001

**Published:** 2013-05-10

**Authors:** Marco Liverani, Jeff Waage, Tony Barnett, Dirk U. Pfeiffer, Jonathan Rushton, James W. Rudge, Michael E. Loevinsohn, Ian Scoones, Richard D. Smith, Ben S. Cooper, Lisa J. White, Shan Goh, Peter Horby, Brendan Wren, Ozan Gundogdu, Abigail Woods, Richard J. Coker

**Affiliations:** 1 London School of Hygiene and Tropical Medicine, London, United Kingdom; 2 London International Development Centre, London, United Kingdom; 3 London School of Economics, London, United Kingdom; 4 Royal Veterinary College, London, United Kingdom; 5 Institute of Development Studies, University of Sussex, Brighton, United Kingdom; 6 Mahidol-Oxford Tropical Medicine Research Unit, Faculty of Tropical Medicine, Mahidol University, Bangkok, Thailand; 7 Oxford University Clinical Research Unit, Hanoi, Vietnam; 8 Centre for the History of Science, Technology, and Medicine, Imperial College London, London, United Kingdom

**Keywords:** emerging diseases, integrated ecology and human health, livestock production, risk characterization, risk management, zoonoses

## Abstract

Background: In many parts of the world, livestock production is undergoing a process of rapid intensification. The health implications of this development are uncertain. Intensification creates cheaper products, allowing more people to access animal-based foods. However, some practices associated with intensification may contribute to zoonotic disease emergence and spread: for example, the sustained use of antibiotics, concentration of animals in confined units, and long distances and frequent movement of livestock.

Objectives: Here we present the diverse range of ecological, biological, and socioeconomic factors likely to enhance or reduce zoonotic risk, and identify ways in which a comprehensive risk analysis may be conducted by using an interdisciplinary approach. We also offer a conceptual framework to guide systematic research on this problem.

Discussion: We recommend that interdisciplinary work on zoonotic risk should take into account the complexity of risk environments, rather than limiting studies to simple linear causal relations between risk drivers and disease emergence and/or spread. In addition, interdisciplinary integration is needed at different levels of analysis, from the study of risk environments to the identification of policy options for risk management.

Conclusion: Given rapid changes in livestock production systems and their potential health implications at the local and global level, the problem we analyze here is of great importance for environmental health and development. Although we offer a systematic interdisciplinary approach to understand and address these implications, we recognize that further research is needed to clarify methodological and practical questions arising from the integration of the natural and social sciences.

## Introduction

Recent studies indicate that more than three-fourths of communicable human diseases are zoonotic in origin (Woolhouse and Gowtage-Sequeria 2005), including diseases associated with significant mortality and morbidity such as avian influenza (H5N1), severe acute respiratory syndrome (SARS), Ebola virus, and Nipah virus. Some of these zoonotic diseases originate in wildlife and others in domestic livestock species; many have the potential to affect humans through a livestock–human interface (Cleaveland et al. 2001) in which domestic livestock can act as “amplifier hosts” (Keesing et al. 2010) for diseases that they contract from wildlife and then pass to humans through frequent and close contact. In light of this, there is growing interest in promoting cooperation between medical and veterinary sectors, as captured in the concept of “one health” (Zinsstag et al. 2011). Scientific research is now focusing on the interactions between humans, domestic animals, and wild animals to better understand the mechanisms of disease emergence and transmission, thus informing more effective policies for communicable disease prevention and control.

In this context, current trends in livestock production have received growing attention (Coker et al. 2011). Although production in Europe and North America has gradually stabilized, in low- and middle-income countries (LIMCs) production is becoming more diverse, with continuing small-scale production—often on farms closely associated with natural habitats and wildlife—as well as highly intensified, industrial-style production units, often in peri-urban settings (Thornton 2010).

It has long been argued that this “livestock revolution” (Delgado et al. 1999) could pose new threats to public health and the environment (Leibler et al. 2009; World Bank 2009). Given our understanding of the links between livestock species and emerging zoonoses, it is relevant to ask whether changes in production systems might increase or decrease the risk of disease emergence in human populations. What husbandry practices are most likely to facilitate or prevent the emergence of new pathogens and their transmission to humans? What can be done to reduce risks? What balance is to be struck between these risks and the health gains from improved nutrition and the economic gains associated with livestock intensification? In this commentary, we provide a brief overview of the diverse range of factors likely to influence zoonotic disease risk, and identify ways in which a comprehensive risk analysis may be conducted by using an interdisciplinary approach. We also present a conceptual framework for interdisciplinary research on this problem, from the identification and analysis of risk drivers to the development of policy options for risk management.

## Intensification and Risk: Ecological and Biological Features

The ecological and biological processes linking livestock production and zoonotic diseases have developed over thousands of years. Domestication of wild animals brought them into close contact with human populations, which themselves were growing more dense as agriculture permitted local population growth, resulting in greater opportunities for transmission and persistence of diseases across humans and domestic and wild animals (Diamond 2002; Wolfe et al. 2007). With subsequent industrialization and urbanization, consumer populations became separated from livestock production, leading to increasingly long supply chains for delivery of animal products and creating new and diverse interactions between humans, livestock, and livestock products.

Over time, growth in demand and production has extended grazing and farming into natural ecosystems, illustrated by the recent transformation of portions of the Amazon forest into grassland for beef production (Fearnside 2005). In many regions, however, expansion of extensive production has approached its limits because of land degradation and lack of available rangelands (Bruinsma 2003). In attempting to meet demand, livestock production has evolved and diversified, with a global trend toward intensification. This trend is characterized by concentration of large numbers of animals in housing units, use of concentrate feed, reduced genetic diversity, vertical integration, and industrial management practices. This “landless” mode of production emerged in the United Kingdom and the United States in the 1930s (Woods 2012) and is now increasingly common in many LMICs, where it is associated with large capital investment, particularly for poultry and pig production (Goss and Burch 2001). While “backyard” and semi-intensive production systems are still widespread, particularly in LMICs, industrial enterprises have been estimated to account for 74% of the world’s poultry production, 40% of pork, and 68% of egg production (Bruinsma 2003).

The health implications of these developments are uncertain. Large-scale intensive units may be good at biosecurity because they are usually more isolated from the external environment and better protected from infection through sustained veterinary control and management procedures. On the other hand, large numbers of susceptible animals in confined spaces increase the risk of disease transmission (Graham et al. 2008; Leibler et al. 2009) and may encourage evolution of pathogens (Mennerat et al. 2010). In Thailand, for example, broiler houses with advanced ventilation systems allow confinement of up to 1 million birds per farm (NaRanong 2008). In such conditions, pathogens are likely to have higher chances of survival, transmission, and rapid evolution (Otte et al. 2007). This can pose serious challenges for biosecurity because complete isolation is unlikely. Pathogens may enter through ventilation units, feed and water systems, or livestock and leave through ventilation, animal products, and production waste (Leibler et al. 2009). Industrial units can produce up to two tons of animal waste every day, which may contain large quantities of pathogens (Hutchison et al. 2005). Much of this waste is often held in large, exposed lagoons, posing an infection risk for wild mammals and birds (Otte et al. 2007).

Other technical innovations associated with intensification have complex risk profiles. Although the widespread use of antibiotics to prevent infection in intensified production may reduce zoonotic risks, nontherapeutic antibiotic use in animal feeds has been shown to serve as a source of antibiotic resistance transferable to human pathogens, where it may affect treatable human infections (Marshall and Levy 2011; Silbergeld et al. 2008; Smillie et al. 2011). The use of imported specialized breeds—characterized by genetic selection toward increased productivity—also has unclear implications for zoonotic risk. Although standardization of specialized breeds may be associated with lower pathogen diversity (Guernier et al. 2004) and thus reduced risk of new pathogen emergence, it can facilitate disease transmission due to homogeneity in genetic susceptibility. In addition, imported breeds may be more vulnerable to pathogens than indigenous breeds, which have unique adaptive traits selected by farmers in local environments over many generations, including resilience to local parasites and diseases (Shand 1997).

In light of these characteristics of modern farming, we suggest that zoonotic risks may be greatest in landscapes where large-scale production units are in close proximity to traditional, small-scale production and to wildlife populations through encroachment of agricultural production into recently deforested natural habitats. Contacts between livestock varieties and wild animals can lead to the introduction of pathogens into intensive production units, where the high density of susceptible animals can facilitate the establishment, transmission, and amplification of pathogens (Gilbert et al. 2006; Slingenbergh et al. 2004).

These conditions are typical of livestock intensification today in many LMICs, and are exemplified by outbreaks of Nipah virus in Asia. The first human outbreaks of Nipah virus, harboured by fruit bats (*Pteropus* spp.), occurred in Malaysia in late 1998, causing > 100 human deaths (Epstein et al. 2006). The index case for these outbreaks, a 30,000-unit intensive pig farm in Malaysia, was established in a deforested area with resident fruit bat populations that frequented fruit trees associated with the farm. The virus was probably passed to the pigs through urine and masticated pellets dropped when the bats fed in overhanging trees. Pigs acted as amplifier hosts, enabling transmission to humans (Pulliam et al. 2012); extensive regional trade of infected animals increased human infections.

## Intensification and Risk: Political and Economic Features

Ecological and biological drivers of disease risk are bidirectionally linked to political and economic factors. Glass and McAtee (2006) identified a range of factors that, although not directly associated with disease emergence, can alter social conditions in a way that creates a new regime of public health risk. For example, public distrust in central government resulting from corruption, conflicts, and political instability may disrupt public health systems and disease surveillance. Such distrust can itself result from zoonotic disease events, as happened in the United Kingdom following the outbreak of bovine spongiform encephalopathy (BSE). The lack of trust in government agencies associated with this outbreak is considered to have been an important factor in the sharp fall in immunizations with the measles, mumps, and rubella vaccine, despite official declarations of their safety (Raithatha et al. 2003).

In the context of agricultural development, the economic conditions in which the intensification of livestock production is taking place can also be an important element in increasing risk. Recent global financial investment in food commodities, development of local and regional chains of capital, and associated changes in national policies affecting the livestock sector have coincided with privatization, market deregulation, and reduced government spending and structural reforms (Steinfeld et al. 2010). In East and Southeast Asia, government subsidies and protective measures have been progressively removed, and producers must compete in an environment increasingly driven by global market forces. In some places, lack of support for producers has been counteracted by the increase in private standards and farm assurance schemes. For example, the Jilin City government in China and a Singaporean company are setting up a food-production area in a 1,450-km^2^ “disease-free” zone that would contain a pig farm expected to eventually process 1 million pigs each year (Kolesnikov-Jessop 2010). However, in less-developed countries, such alternative forms of risk regulation may be absent.

What are the effects of these economic changes on zoonotic risk? At each transitional stage in the value chain, from farm to final market, attempts to increase or maintain profit margins may create opportunities for risks to develop (Mcleod et al. 2009). As new large retail units seek to capture market shares, they must offer competitive prices by cutting costs. Pressure to survive in a highly competitive market with minimal government support and lack of adequate regulatory oversight may encourage retailers and producers to engage in risky practices, including disinvestment in animal health and biosecurity.

Other effects may be less direct. For example, attempts to decrease labor costs may result in the less-effective exercise of local control measures, as low-level workers develop labor- and time-saving practices for dealing with management pressure and working conditions. Withdrawal of state subsidies may also affect smallholders (family farmers) who are tied to large agribusiness companies through contract farming (Catelo and Costales 2008). Large companies often provide contractors with technical support and veterinary assistance; however, some may not source supply from smallholders if they can find larger producers who are able to make the necessary investments to operate profitably (Costales et al. 2006). Under these circumstances, the burden is on the smallholders to make the investments. If they do not, they may be taken out of the market chain and potentially turn to illegal trade. Indeed, illegal movement of animals and derived products represents one of the most important sources of spread of infectious diseases. One example is the significance of illegal trade in poultry between countries in Southeast Asia in the continued spread of avian influenza H5N1 across that region (Pfeiffer et al. 2011).

Compared with small-scale producers, large producers may gain political power and influence, thus making governments less likely to impose strong regulations that affect their interests. For example, Wallace (2009) concluded that agribusiness companies in Thailand had a great influence on policy makers during the first outbreaks of avian influenza, to the extent that industrial units accelerated production despite public health risks. In the early phases, an initially slow response by government agencies exacerbated the problem, although by most accounts Thailand later became an exemplar in the region of avian influenza control through active surveillance and quick intervention (Safman 2009).

Finally, international trade is another element in the political economy of risks from livestock production. Global trade in livestock and livestock products has substantially increased in recent years because of the proliferation of free trade agreements, more efficient transport and communication systems, and intensive agriculture (Steinfeld et al. 2010). Although this development has promoted the adoption of international standards and regulations—such as the standards set by the World Organization for Animal Heath—it has also enhanced the opportunities for widespread transmission of viral infections and bacterial contaminants in ways that are still poorly understood (Hodges and Kimball 2005).

## From Risk Drivers to Risk Management

**Figure 1:**
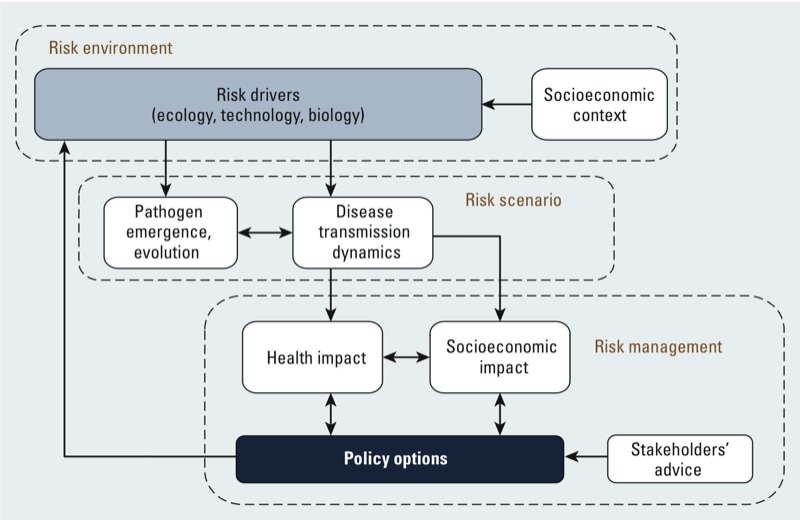
A conceptual framework that can guide interdisciplinary research on zoonotic risk in the new livestock industries. Each rectangle with a dotted border represents a level of analysis in the research program. The first step requires the characterization of risk environments, including an understanding of ecological and biological risk drivers, as well as the wider socio­economic contexts that influence them. The second step entails the production of plausible risk scenarios that explore the ways in which risk environments may influence pathogen emergence, evolution, and transmission to human population. Finally, the third level of analysis evaluates the health and economic burden for given risk scenarios in order to identify policy options for risk management, in collaboration with stakeholders and agricultural experts.

The brief overview of risk factors that we have presented here highlights the multidimensional nature of the links between changing livestock production practices and the emergence and spread of zoonotic diseases. Given this complexity, understanding the conditions that create zoonotic risk requires a research approach that links both animal and public health sectors and natural and social sciences. Other researchers have stressed the need for integration in this area (Brownlie et al. 2006; Dry and Leach 2010; Parkes et al. 2005; Scoones 2010; Wilcox and Kueffer 2008). However, few efforts have been made to translate the research agenda into novel methods and concepts that can sustain and guide empirically informed scientific work (Waltner-Toews et al. 2008; Wilcox and Gubler 2005; Wood et al. 2012). In contributing to these efforts, we suggest that interdisciplinary research on zoonotic risk should be able to account for the complexity of “risk environments” (Barnett and Blaikie 1992), rather than simple linear causal relations between risk drivers and disease emergence and/or spread. From this perspective, the key scientific challenges are *a*) to correctly define and understand the risk environment as part of a larger system that creates risk; and *b*) to deploy a method that combines insights along the full extent of the chain of evidence from viral genome sequencing to animal keeping and on to animal market economics and the politics of food industry regulation. To address such complex interactions and develop effective policy, we need integrated research that operates at different levels of analysis, from the study of risk environments to the development of risk scenarios and identification of policy options for risk management. These linked levels constitute a conceptual model illustrated in Figure 1.

The first level of analysis involves integration of disciplinary expertise to provide a rich sociobiological characterization of risk environments. The contribution of social scientists enhances understanding of both the political economy and the actual practice of livestock production and, above all, the relations between these. In studies to date, analysis of livestock industries is often limited to an aggregate of statistical values, framed within broad categories such as “industrial,” “semi-intensive,” “backyard,” or “mixed farming” production systems (Robinson et al. 2011). Although such classifications are useful for organizing the collection and comparison of data for analysis, they inevitably obscure the complexity of an economic sector that is becoming increasingly differentiated. In turn, improved knowledge of the structure and practices of livestock production can provide a framework for strategic pathogen sampling, allowing the linkage of production systems—with their microbial and genetic profiles—to identify vulnerabilities and drivers of pathogen diversity and evolution at each stage of the production process.

A second level of analysis is needed to explore the ways in which such sociobiological configurations may influence pathogen emergence, evolution, and transmission into human populations, including the development of plausible risk scenarios. Types of zoonotic pathogens range from those that can infect humans but do not spread, to pathogens that spread easily between humans (Wolfe et al. 2007); evolutionary or ecological conditions that favor emergence of the latter types pose the greatest risk. Mathematical modeling can provide insights into how ecological and evolutionary processes leading to emergence and spread of new zoonotic pathogens are likely to be affected by changes in specific features of the risk environment. There is a growing body of modeling and empirical research on pathogen emergence and evolution, as well as on pathogens’ dependence on contact networks and routes of transmission (Antia et al. 2003; Lloyd-Smith et al. 2009; Read and Keeling 2003, 2006). Such models must consider three key processes, all of which are likely to be affected by livestock production systems and their interactions:

The initial generation of genetic novelty, which may vary between production systems. For instance, differences in pathogen diversity and antibiotic use in production systems may lead to different levels of genetic recombination (MacLean et al. 2010).
Subsequent stepwise adaptation of novel, more virulent pathogen types to one or more host species (Antia et al. 2003), which will be affected by different animal life spans and different contact patterns within and between host species.
Sustained transmission within the new host population(s), which will be influenced by contact patterns, genetic susceptibility, and local population sizes and densities, which will affect the chance of local extinctions of novel pathogens.

Recent advances in molecular epidemiology can also provide powerful tools for testing predictions of how pathogens spread and evolve, through the capacity to characterize pathogen genomes for similarities and differences across animal and human populations (Parkhill and Wren 2011).

Finally, a third level of analysis is required to estimate the health and economic burden for given risk scenarios and identify policy options for risk management. This complex issue also requires integration of disciplinary perspectives and diverse empirical material, including *a*) epidemiological models at the population level, *b*) health systems indicators, *c*) inward and outward trade indices, *d*) information on existing health and agricultural policy and regulations, *e*) cultural attitudes toward specific interventions, *f* ) political constraints and opportunities, and *g*) economic data on livestock production systems. After the epidemiological models have been completed, macroeconomic modeling has the potential to embed such heterogeneous data sets into a comprehensive framework for assessments of specific interventions in terms of cost benefits and cost effectiveness (Beutels et al. 2008). For example, the computable general equilibrium (CGE) approach, a member of the “whole economy” class of economic models, can be used to account for differential effects of policy options on categories of social actors (e.g., consumers, producers, households, governments) (Smith et al. 2005). In addition, combined with outputs from epidemiological models, the CGE approach can accommodate the fact that zoonotic disease and associated control initiatives will affect the economy and population health over time, and that the time profiles of costs and benefits will be sensitive to the sequence of events and interventions (Smith et al. 2009). However, such models must be sufficiently flexible and open to account for the variety of qualitative insights on cultural attitudes and governance constraints, in addition to quantitative data on macroeconomic trends and health indicators (Smith et al. 2011). The advice and experience of agricultural experts and other stakeholders must also be included as an active element of the risk management strategy.

## Conclusions

Livestock intensification will characterize global development for decades to come as countries lift themselves out of poverty. There are major potential consequences of this change, not only with respect to emerging zoonotic diseases but also to agricultural sustainability, food security, and climate change effects (Friel et al. 2009). It is not at all clear that intensification will lead to a greater frequency of zoonotic disease emergence, although some important risk factors are present. What is needed is an approach to understanding where and how risk can be generated. This requires an intersectoral and interdisciplinary approach and a stepwise process leading from improved knowledge and specification of risk drivers to policy options for risk management. However, integrated research like this has particular methodological challenges. For instance, the development of modeling tools able to account for the combination of potential risk factors, as well as their health and economic effects, requires the translation of a diverse range of socioeconomic, biological, cultural, and epidemiological information into a common language for data collation, comparability, and analysis. This process is likely to be difficult because different disciplines may rely on different bases for causal inference, notions of impact, measurement systems, and theoretical frameworks. Thus, continued disciplinary interactions, mediated by boundary-spanning concepts such as risk environment, are crucial to refine research questions and methods toward a common goal. Fruitful relationships of this sort have led to novel insights on the dynamics of epidemics—for example, the role of concurrent sexual relationships in the context of HIV transmission (Morris et al. 2009).

Broad interdisciplinary approaches are needed to better understand the complex interactions of factors that act together to increase or reduce risks to animal and human health in the new livestock industries. However, such a research program demands solutions to theoretical and practical issues that are rarely addressed in policy statements on “one health.” These issues are likely to gain more relevance as the focus moves from the domain of policy making to the research needed to inform policy. Thus, these issues should become more central in future studies of emerging diseases, as in other contexts of interdisciplinary integration between the natural and social sciences.
